# Individualist–Collectivist Differences in Climate Change Inaction: The Role of Perceived Intractability

**DOI:** 10.3389/fpsyg.2019.00187

**Published:** 2019-02-12

**Authors:** Peng Xiang, Haibo Zhang, Liuna Geng, Kexin Zhou, Yuping Wu

**Affiliations:** ^1^School of Social and Behavioral Sciences, Nanjing University, Nanjing, China; ^2^School of Government, Nanjing University, Nanjing, China; ^3^Nanjing Institute of Environmental Sciences, Ministry of Environmental Protection, Nanjing, China

**Keywords:** climate change inaction, perceived intractability, climate change, collectivism, individualism

## Abstract

The willingness to take action against climate change may be shaped by cultural orientations. The present study investigated individualist–collectivist differences in climate change inaction as well as the mediating role of perceived intractability. In Study 1, a survey of 182 undergraduates showed that greater perceived intractability of climate change was significantly related to a lower frequency of climate-friendly actions in the preceding 6 months. In Study 2, participants who were exposed to information concerning the intractability of climate change (experimental group, *n* = 98) reported a significantly greater perceived intractability of climate change and lower intention to assume a low-carbon lifestyle than those presented with neutral information (control group, *n* = 83). Based on Studies 1 and 2, participants with collectivist or individualist orientations were recruited from a pool of Chinese undergraduate students in Study 3. We found that participants with a more individualist orientation (*n* = 62) are more subject to perceived intractability, and less likely to take climate-friendly action than those with a more collectivist orientation (*n* = 94), and individualist/collectivist status affects climate change inaction through perceived intractability as mediator. The implications of these findings are discussed in relation to the promotion of public engagement with climate change by mitigating perceived intractability.

## Introduction

Despite increasing pressure to deal with climate change, individuals have been hesitant to respond effectively. As stated by Pölzler (2015), “we consume as much as we always did, drive as much as we always did, eat as much meat as we always did.” Indeed, public opinion polls and previous studies have also indicated that our action on climate change is limited. According to the results of a Eurobarometer survey carried out in 2009, just over half (53%) of European Union citizens say they took some kind of action to combat climate change over the previous 6 months (European Commission, 2009). Likewise, Fu et al. (2015), in a survey of 2,100 respondents, found that only 57.05% of Chinese individuals reported always or often engaging in energy-saving activities to alleviate climate change. Now that climate change is one of the greatest challenges facing global society, why are we so reluctant to take action against it? Recently, individual inaction over climate change mitigation, so-called climate change inaction, has been attracting increasing attention.

### Climate Change Inaction and Its Psychological Barriers

Though climate change inaction has appeared periodically in public discourse and scientific literature, a clear definition is still lacking. Roughly, any form of an actor’s inactive state in addressing climate change could be described as climate change inaction. The concept of “climate change inaction” is widespread in the environmental economic literature. This line of inquiry aims to calculate the cost of climate change inaction versus inputs caused by action on climate change (e.g., Rodríguez-Labajos, 2013). Recently, this concept was introduced into the field of environmental psychology in order to address the question of why we do not take action on climate change. Climate change inaction occurs at various levels involving individuals, businesses, and governments. Individual inaction on climate change may be manifested in indifference to climate change in daily routine, e.g., denying climate change as a vital issue (Engels et al., 2013; Liu, 2015) or continuing a high-carbon lifestyle (Boucher, 2016). On a macro level, psychological (e.g., short-termism) and structural (e.g., restricted financial resources) factors could hinder companies and governments in actively responding to climate change (Slawinski and Bansal, 2012; Slawinski et al., 2015; Finke et al., 2016). Obviously, forms of climate change inaction intersect at different levels. For instance, governmental non-supportive policy will discourage companies’ investment in climate-friendly products, which will in turn foster consumers’ climate change inaction.

In the present article, we focus on individual climate change inaction. For most of us, one of the most feasible ways to tackle climate change may lie in low-carbon lifestyle changes. Clearly, individuals face a wide range of options. For example, Wynes and Nicholas (2017) suggested that annual personal carbon emissions could be reduced by 0.8t via eating a plant-based diet. Despite the feasibility and efficiency of low-carbon lifestyles, many of us still engage in behaviors that are detrimental to the environment or fail to engage in ameliorative actions.

Traditionally, conventional wisdom ascribes individual inaction against climate change to attitudinal deficit, i.e., people tend to underestimate or deny the threat posed by climate change. Of fundamental interest to researchers is determining how and why we perceive climate change as we do. Research related to climate change risk perception has indicated that people are inclined to perceive climate change as a psychologically distant risk that might occur far in the future, impacting distant places and affecting people dissimilar to themselves (Leiserowitz, 2004; Jones et al., 2017). Other factors that sway climate change risk perceptions were also documented by several researchers, e.g., age, personal experience, and cultural worldview. The underlying assumption behind these studies is that climate-friendly behavior could be nudged by revising individuals’ perceived risk. However, this assumption has not been consistently confirmed (e.g., Safi, 2011).

Unlike the above-mentioned research, climate change inaction research places a direct emphasis on the circumstances in which people stay inactive in the face of climate change. This research argues that we must first clarify the causes of climate change inaction in order to overcome it (Pölzler, 2015). There is a large body of researchers attempting to identify the constraints of climate change inaction. Among these, psychological barriers have attracted much attention, as they are more susceptible to interventions than are other factors (Stern, 2011; Swim et al., 2011). A number of psychological (as opposed to structural) barriers have been proposed (Lorenzoni et al., 2007; Johnson and Levin, 2009; Moser and Ekstrom, 2010; Gifford, 2011; Mäkiniemi and Vainio, 2014; Gifford and Chen, 2017). Utilizing a mixed-method approach, Lorenzoni et al. (2007) identified a range of barriers that members of the United Kingdom public perceive to engaging with climate change, i.e., uncertainty, skepticism, and reluctance to change one’s lifestyle. Likewise, Gifford and Chen (2017) demonstrated that three kinds of psychological barriers are significantly associated with fewer climate-ameliorative food choice intentions (e.g., purchase organically grown food, eat less meat): denial (e.g., “There’s no need to make these changes because I’m not convinced that a serious environmental problem even exists”), conflicting goals and aspirations (e.g., “I’m concerned that these changes will take up too much of my time”), and tokenism (e.g., “I’m satisfied with my current way of doing things”).

### Individualism–Collectivism and Climate Change Inaction

As mentioned above, barriers to climate change action have been attracting increasing attention, but few studies have explored how cultural orientation may result in climate change inaction. There is a wealth of literature concerning climate change, demonstrating that cultural orientation plays a crucial part in an individual’s attitudinal response to climate change (e.g., Shi et al., 2015; Xue et al., 2016). For instance, a large body of research drawing on Cultural Theory (Douglas and Wildavsky, 1982) has well documented that the ways in which people perceive climate change risk are shaped by the cultural worldviews that they hold (Weber, 2010; McNeeley and Lazrus, 2014). However, individual action against climate change does not necessarily depend on our risk perception of climate change (Bain et al., 2016). Certainly, there are studies of behavioral responses to climate change, but most of them have largely focused on the difficulty of implementing certain practices rather than on the phenomenon of climate change inaction itself (Slawinski et al., 2015). The results of a study conducted in Kiribati by Kuruppu (2009) suggested that people’s capacity to diversify water resources (a way of adapting to climate change) is constrained by cultural values attached to the material resources they possess. Still, we cannot infer that cultural processes cause climate change inaction based on Kuruppu’s findings.

Given that individual responses to climate change could be shaped by cultural orientation, it is reasonable to assume the reverse-that is, climate change inaction may also vary as a function of certain cultural factors. We believe that individualism-collectivism might be one such factor. A great deal of research has utilized individualism–collectivism as a key dimension to analyze and differentiate cultural orientation. According to the existing research, individualism (vs. collectivism) is characterized by the view of an independent self (vs. interdependent self) (Markus and Kitayama, 1991). Specifically, individualists focus on personal autonomy and individual uniqueness and place personal goals over group goals. By comparison, collectivists care about group norms and collective harmony and subordinate personal goals to the group goals (Wagner and Moch, 1986; Strunk and Chang, 1999; Voronov and Singer, 2002). Though most works related to individualism–collectivism have been cross-cultural (e.g., differences in individualism/collectivism across countries), there is some evidence to suggest that a distinction between individualism and collectivism can exist within a single culture in the form of individual differences (Moorman and Blakely, 1995). Following this, the current investigation focuses on individualism–collectivism at the individual, psychological level.

We propose that individualism is more related to climate change inaction than is collectivism. This assumption derives support from the following considerations. First, individualist versus collectivist orientations have been found to influence pro-environmental behavior (Cho et al., 2013). Roughly, collectivist individuals are more likely to engage in a variety of pro-environmental behaviors than are those with individualist tendencies, including resource conservation (Dunlap and Liere, 1984) and green purchasing behaviors (Kim, 2005). Further, a survey conducted in New Zealand by Semenova (2015) found that the more environmentally active group (sampled from the sustainable communities) was more collectivist in its value orientation than was the less environmentally active group (sampled from the general population). Similar findings were also reported by Jia et al. (2017), who demonstrated that environmental activists were more likely to endorse self-transcendent values (similarly to collectivism, e.g., universalism-concern), while non-activists were more likely to endorse self-interest values (similarly to individualism, e.g., self-direction). In addition, several studies within the framework of cultural worldview have also suggested that individualist worldviews are negatively related with concern about climate change, willingness to behave in climate-friendly ways, and acceptance of related policy measures (e.g., Xue et al., 2016).

### Perceived Intractability, Individualism–Collectivism, and Climate Change Inaction

In addition to the individualism–collectivism difference in climate change inaction, we seek to further demonstrate that this difference might be caused by individuals’ perceptions of the intractability of climate change. To the best of our knowledge, the concept of intractability in the environmental psychology domain was originally developed by Campbell (1983) as one of the five characteristics of ambient stressors. According to Campbell (1983), intractability refers to objective features of ambient stressors, where the isolated efforts of individuals do not bring about substantial changes in the presence of ambient stressors. The fact that climate change is a global problem that requires the combined actions of millions of individuals working together has been accepted by the international community. However, this fact also yields an objective dilemma, that is, climate change is intractable to the action of individuals to change it. Therefore, perceived intractability of climate change in the present study refers to one’s belief that climate change cannot be addressed by individual action. As an intrinsic characteristic of climate change, intractability is distinguished from other closely related concepts, such as climate change helplessness (Salomon et al., 2017). Climate change helplessness was defined by Salomon et al. (2017) as the belief that climate change is beyond personal control, which emphasizes individual experience instead of climate change.

Given that individual actions or the collective actions of many individuals cannot ameliorate climate change within a limited space-time frame, it appears intuitively reasonable that individuals who realize the intractability of climate change are likely to be inactive against climate change. Taking carbon emissions as an example, a recent report on the Global Carbon Budget, 2017 published by the Global Carbon Project (GCP) claimed that global CO_2_ emissions in 2017 reached a new record of 36.8 Gt (Global Carbon Budget, 2017). Although many actions with the potential to reduce annual personal emissions have been recommended, e.g., living car-free (2.4 tCO_2_e saved per year), avoiding airplane travel (1.6 tCO_2_e saved per roundtrip transatlantic flight), and eating a plant-based diet (0.8 tCO_2_e saved per year) (Wynes and Nicholas, 2017), emissions reductions realized by individual lifestyle changes still seem rather trivial. In this case, it is possible that individuals may consider climate change an intractable issue, since their low-carbon behavior is ineffective or meaningless for climate change, which in turn limits their action on climate change.

Furthermore, individualism and collectivism may relate to different propensities to perceive climate change as intractable. Individualism and collectivism may involve the perceived intractability of climate change in two ways. In the first, when confronted with climate change, individuals with individualist orientations usually rely on a self-reliant coping style, that is, viewing behavioral changes related to climate change as a personal matter and tackling climate change on their own. In this case, individualists may feel that climate change is so intractable that it cannot be solved by their individual actions. By contrast, when confronted with climate change, collectivists may regard fighting climate change as a collective mission, and thus, although individual contributions are small, they still matter. Hence, it is less likely for collectivists to perceive climate change as intractable. As a second example, it has been found that individualism and collectivism differ in social desirability: collectivists are more likely to be socially desirable than individualists (Lalwani et al., 2006; Riemer and Shavitt, 2011; Oh, 2013). Given that members of society are expected to take action against climate change, it is evident that collectivists are more responsive to this social expectation than individualists. In this case, in order to maintain a positive and normative image, collectivists are less prone to acknowledge that climate change is intractable.

## Overview of the Present Research

Based on the above descriptions, the present research aimed to demonstrate individualism–collectivism differences in climate change inaction and test whether the perceived intractability of climate change could account for this phenomenon. To this end, three studies were conducted. Given that the relationship between perceived intractability and climate change inaction has thus far received little empirical support, the first two studies were conducted to provide initial evidence for it in the general population. The third study specifically investigated individualism–collectivism. Specifically, Study 1 was designed to identify the possible correlation between perceived intractability and climate change inaction. We hypothesized that climate change inaction is correlated positively with perceived intractability. The aim of Study 2 was to provide more convincing evidence for the causal effect of perceived intractability on climate change inaction. We expected that participants who were reminded of the intractability of climate change would show a greater tendency to climate change inaction than participants in the control condition. In Study 3, we investigated and compared perceived intractability and climate change inaction among collectivist and individualist participants. We hypothesized that collectivist participants would perceive higher intractability than individualist participants, and thus be less likely to take climate-friendly action.

It is noted that because other factors (e.g., beliefs, risk perception, or knowledge related to climate change) have been shown to relate to action on climate change (Ohe and Ikeda, 2005; Hidalgo and Pisano, 2010; Vainio and Paloniemi, 2013; Shi et al., 2015; Mase et al., 2016), we tested these factors in conjunction with the variable of the perceived intractability of climate change. We expected that climate change inaction is related to the perceived intractability of climate change, even when controlling for other related variables.

## Study 1

### Methods

#### Participants

In total, 182 undergraduates (56% males) participated in this survey. Their ages ranged from 17 to 24 years (*M* = 18.79, *SD* = 1.46). Participants were recruited from a large subject pool from Nanjing University. They received a small gift (worth around 5 USD) for their participation.

#### Procedure and Materials

Participants were asked to access the online survey and complete a series of questionnaires via Wenjuanxing, a Chinese online survey website. In addition to perceived intractability, two other related variables were measured: belief in climate change and climate change risk perception. The measures, which were developed based on English scales, were translated into Chinese so that respondents could understand them. Equivalence between the Chinese and the English versions was ensured through careful checking and back-translation.

##### Belief in Climate Change (BCC)

Belief in Climate Change (BCC) was measured with the three-item scale developed by Vainio and Paloniemi (2013). This scale asked participants if they agreed or disagreed with three statements: “Climate change is an unstoppable process; we cannot do anything about it,” “The seriousness of climate change has been exaggerated,” and “Emission of CO_2_ (carbon dioxide) has only a marginal impact on climate change.” Participants were asked to answer on a 4-point scale from 1 (*totally agree*) to 4 (*totally disagree*). Cronbach’s α for this scale was 0.608.

##### Climate Change Risk Perception (CCRP)

The Climate Change Risk Perception (CCRP) was a measure based on Leiserowitz’s (2006) Climate Change Risk Perception Index. The measure includes nine items rated on a 4-point scale, with higher scores indicating that participants display greater CCRP. A sample item is “How concerned are you about climate change?” Cronbach’s α for this scale was 0.864.

##### Perceived Intractability of Climate Change (PICC)

A 4-item Likert-type scale was developed to measure Perceived Intractability of Climate Change (PICC). PICC was operationalized with the following items: (1) my individual action would likely do little to aid the fight over climate change; (2) I can bring a fundamental change in climate change in everyday life; (3) climate change couldn’t be relieved by my day-to-day behavior; and (4) my daily action could have a positive impact on climate change. Participants were asked to answer on a 4-point scale from 1 (*totally agree*) to 4 (*totally disagree*). The second and fourth items were reverse-scored so that high scores represented a high perceived intractability. Cronbach’s α for this scale was 0.758.

##### Climate Change Inaction (CCI)

Climate Change Inaction (CCI) was measured with one straightforward question: “How often, on a scale of 1 (*not very often*) to 7 (*very often*), have you taken some kind of action to combat climate change over the last 6 months?” The values were reversed so that high values represented high climate change inaction.

### Results

All data analyses were performed with SPSS 20.0. We first computed descriptive statistics, and then explored the relationship between PICC and CCI with hierarchical multiple regression.

The means, standard deviations, and Pearson correlations of the variables are presented in Table 1. We found that CCI was significantly related to PICC (*r* = 0.476, *p* < 0.01). That is, higher perceived intractability of climate change was related to lower frequency of climate-friendly behavior over the past 6 months. In addition, there was a significant negative correlation between CCI and BCC (*r* = -0.269, *p* < 0.01), as well as CCRP (*r* = -0.227, *p* < 0.01).

**Table 1:** Descriptive statistics and correlations for major variables of Study 1.

	*M*	*SD*	*Max*	*Min*	1	2	3	4
1. BCC	8.92	2.06	12.00	3.00				
2. CCRP	23.79	5.16	36.00	12.00	0.044			
3. PICC	12.43	5.43	16.00	4.00	–0.413^∗∗^	–0.187		
4. CCI	3.39	2.01	7.00	1.00	–0.269^∗∗^	–0.227^∗∗^	0.476^∗∗^	


*BBC, Belief in Climate Change; CCRP, Climate Change Risk Perception; PICC, Perceived Intractability of Climate Change; CCI, Climate Change Inaction. ^∗^*p* < 0.05; ^∗∗^*p* < 0.01.*

Using a hierarchical multiple regression analysis, we tested our hypothesis that individuals who rated climate change as more intractable less frequently took climate-friendly action in the preceding 6 months. In the model, CCI was treated as the dependent variable, and BCC and CCRP were entered into the first block as control variables, and PICC score into the second block. The results (see Table 2) indicated that CCI was significantly negatively related to BCC (β = -0.260, *p* < 0.01) and CCRP (β = -0.216, *p* < 0.01). The results also indicated that after controlling the effect of BCC and CCRP, the effect of PICC on CCI was still statistically significant (β = -0.151, *p* < 0.01).

**Table 2:** Summary of hierarchical multiple regression analyses for predicting CCI of Study 1.

	Model 1		Model 2	
Variables	*B* (*SE*)	β	*B* (*SE*)	β
BCC	–0.252 (0.068)	–0.260^∗∗^	–0.090 (0.069)	–0.093
CCRP	–0.084 (0.027)	–0.216^∗∗^	–0.057 (0.026)	–0.146^∗^
PICC			0.151 (0.027)	0.410^∗∗^
*R*^2^		0.119^∗∗^		0.253^∗∗^


*BBC, Belief in Climate Change; CCRP, Climate Change Risk Perception; PICC, Perceived Intractability of Climate Change; CCI, Climate Change Inaction. ^∗^*p* < 0.05; ^∗∗^*p* < 0.01.*

### Discussion

Since no study has been conducted so far to explore the relationship between CCI and PICC, Study 1 aimed to provide preliminary evidence for this issue. Clearly, the results of Study 1 showed that CCI, which is operationalized as a low frequency of climate-friendly action over the preceding 6 months, is significantly correlated with the level of PICC. This correlation remains significant even when we control for other related variables, such as BCC and CCRP. These findings support our hypothesis that climate change inaction is correlated positively with perceived intractability. However, it is still unclear whether an activation in PICC salience would result in CCI. We would like to further examine the causal effect of PICC on CCI experimentally. In addition, as CCI in Study 1 was operationalized as a retrospective evaluation of past behavioral patterns, such self-reported results may be swayed by recall bias whereby participants are indifferent to their past behaviors. Therefore, prospective intention (rather than retrospective frequency) relating to climate-friendly actions was used alternatively in Study 2. Another potential deficiency is the knowledge deficit, that is, participants do not know which daily behaviors are helpful in curbing climate change. This problem was avoided in Study 2 by assessing the participants’ knowledge of climate change action (KCCA).

## Study 2

### Methods

#### Participants

One hundred and ninety-eight undergraduates initially volunteered to participate in the study, recruited from a large subject pool from Nanjing University. All participants were evenly distributed to the experimental or control group. Seventeen cases were deleted for not returning their questionnaires. Thus, the final sample was based on a total of 181 participants (experimental group: *n* = 98; control group: *n* = 83). Their mean age was 20.80 (*SD* = 1.98), and 51% of them were females. They were paid 30 CNY (around 5 USD) for their participation.

#### Procedure and Materials

Participants were asked to complete the survey experiment via Wenjuanxing. The experimental procedure was as follows. First, given that Study 1 showed that BCC and CCRP are potential contributors to climate change inaction, the experimental and control groups first completed the BCC and CCRP instruments, which were the same as those used in Study 1, before receiving subsequent interventions. We aimed to test whether the baseline levels of BCC and CCRP were similar between the experimental and control groups in order to control for their confounding effect on CCI. Second, participants read a passage about climate change, which served to manipulate the PICC variable. Specifically, the experimental group was presented information indicating climate change is inherently intractable, whereas participants in the control group were presented with neutral information describing the manifestations of climate change, i.e., global warming, acid rain, and ozone depletion (see Appendix 1). After reading these distinct passages, both groups reported their perceived intractability of climate change as in Study 1. Third, all participants finished the measurement of CCI. Unlike Study 1, a novel measure of CCI was employed in the present study. Considering that individuals’ climate change (in)action may depend on the level of knowledge about climate-friendly behaviors, we created a questionnaire to measure participants’ KCCA. Participants were presented with a checklist containing ten daily behaviors. Sample items include “Cut meeting times as short as possible,” “Eat less meat if you can,” and “Machine-wash clothing only when there is a full load.” We instructed them to identify whether each item in their view is climate-friendly with yes or no (yes = 1, no = 0). As a matter of fact, all the items are climate-friendly. We calculated the measure of KCCA by adding the participants’ answers, so that higher scores indicate higher levels of KCCA. Participants were then asked to report how likely they were to perform their chosen behaviors in the future on a 7-point Likert-type scale ranging from 1 (*highly unlikely*) to 7 (*highly likely*). The values were reversed so that high values represented high CCI.

### Results

Table 3 presents the descriptive statistics. An independent-samples *t*-test was performed on PICC between the experimental and control groups. Consistent with the manipulation, there was a significant difference for PICC between the experimental and control groups, with the experimental group showing higher scores: *t*(179) = -5.48, *p* < 0.001, *M*_d_ = -3.85, 95% CI = [-5.23, -2.46], Cohen’s *d* = -0.82. Furthermore, the results showed that the CCI scores of the experimental group were significantly higher than those of the control group [*t*(179) = -4.86, *p* < 0.001, *M*_d_ = -0.91, 95% CI = [-1.28, -0.54], Cohen’s *d* = -0.73]. An ANCOVA was conducted to assess between-group differences in CCI, with BCC, CCRP, and KCCA as co-variates. The effect of group remained significant for CCI [*F*(1,176) = 5.649, *p* < 0.001, $\eta_{p}^{2}$ = 0.111]. This result showed that the difference in PICC and CCI between the two groups was independent of BCC, CCRP, and KCCA.

**Table 3:** Descriptive statistics and independent samples *T-*test for major variables of Study 2.

	Experimental group		Control group		*Max*	*Min*	*t*
	*M*	*SD*	*M*	*SD*			
BCC	8.98	2.21	8.65	2.16	12.00	3.00	–1.009
CCRP	26.14	6.08	26.41	5.40	36.00	9.00	0.310
KCCA	4.91	2.47	4.75	2.12	10.00	1.00	–1.253
PICC	14.52	5.58	10.67	3.80	16.00	4.00	–5.483^∗∗^
CCI	3.48	1.56	2.57	0.94	7.00	1.00	–4.861^∗∗^


*BBC, Belief in Climate Change; CCRP, Climate Change Risk Perception; KCCA, Knowledge of Climate Change Action; PICC, Perceived Intractability of Climate Change; CCI, Climate Change Inaction. ^∗^*p* < 0.05; ^∗∗^*p* < 0.01.*

### Discussion

Participants who had just been exposed to intractability-inducing information reported higher levels of perceived intractability of climate change than those exposed to neutral information, whereas the differences in other related variables (i.e., BCC, CCRP, and KCCA) between the experimental and control groups were not statistically significant. Moreover, participants in the experimental group were more likely than those in the control group to exhibit climate change inaction, i.e., being reluctant to perform climate-friendly action. Taken together, these results support our second hypothesis, which postulated that those who were reminded of the intractability of climate change would be less likely to take climate change action than those in the control condition.

In short, Study 2 provided preliminary evidence for the causal effect of perceived intractability on climate change inaction. This finding further ruled out the possibility that perceived intractability may be used as self-justification by individuals to defend their inaction on climate change. Thus Study 2 provided the preconditions for Study 3, which examined the effect of individualistic/collectivistic status on climate change inaction and the mediation of perceived intractability.

## Study 3

### Method

#### Participants and Procedure

The initial sample included 788 freshmen (148 women, 640 men; age ranging from 17 to 23) from Zhejiang Ocean University. Participants were initially recruited for the Chinese Values Changing Survey (CVCS) conducted in August 2017, which was planned to trace changes in values annually from 2017 to 2020 among Chinese adolescents. All participants were instructed to finish a battery of questionnaires online at the time of the survey, including an individualism–collectivism measure. Specifically, individualism–collectivism was assessed by a 16-item scale. This version of the scale was derived from the Individualism–Collectivism Scale (Singelis et al., 1995), which differentiates between horizontal and vertical types of individualism and collectivism, and the items related to horizontal individualism (e.g., “I often do ‘my own thing”’; Cronbach’s α = 0.744) and collectivism (e.g., “I feel good when I cooperate with others”; Cronbach’s α = 0.717) were used. The participants responded to 16 items on a 9-point Likert scale ranging from 1 (*strongly disagree*) to 9 (*strongly agree*).

Chinese Values Changing Survey provided a starting point for the Study 3, which was conducted in October 2017. Because of our conceptualization of individualism–collectivism as an individual-difference variable, we only selected participants with extreme individualist or collectivist orientations for Study 3. Following a previously documented procedure (for greater detail, see Caldwell-Harris and Ayçiçegi, 2006), 227 participant candidates were picked and identified as either individualists (*n* = 96) or collectivists (*n* = 131) according to their initial scores on the individualism–collectivism measure. ^1^ All participant candidates received an email inviting them to undertake the survey. In addition to the variables assessed in Study 2, the identical measure of individualism–collectivism was also included. A total of 156 surveys were completed and returned (67% response rate), with participants in both an individualist group (*n* = 62) and a collectivist group (*n* = 94). Therefore, the final sample of Study 3 consisted of 156 freshmen (43 women, 113 men), with a mean age of 18.85 years (*SD* = 1.458). Participants received an honorarium of 30 CNY for participating in the study.

### Results

The means, standard deviations, and Pearson correlations of the variables are presented in Table 4. An independent-samples *t-*test was performed on the various measurements between the individualist and collectivist groups (see Table 5). As expected, the scores on the individualism dimension of the individualist group were significantly higher than were those of the collectivist group: *t*(154) = 5.77, *p* < 0.001, *M*_d_ = 8.22, 95% CI = [5.40, 11.03], Cohen’s *d* = 0.95. Similarly, scores on the collectivism dimension of the individualist group were significantly lower than were those of the collectivist group, *t*(154) = -6.31, *p* < 0.001, *M*_d_ = -10.61, 95% CI = [-13.95, -7.28], Cohen’s *d* = -1.04. No significant difference was found in BCC, CCR, or KCCA between groups (*p >* 0.05).

**Table 4:** Descriptive statistics and correlations for major variables of Study 3.

	*M*	*SD*	*Max*	*Min*	1	2	3	4	5
1. BCC	8.65	2.10	12.00	3.00					
2. CCRP	24.40	5.30	36.00	12.00	–0.004				
3. KCCA	4.78	2.22	10.00	1.00	0.250	–0.002			
4. PICC	11.99	4.71	16.00	4.00	–0.333^∗∗^	–0.120	–0.205		
5. CCI	2.73	1.34	7.00	1.00	–0.321^∗∗^	–0.160^∗^	–0.133	0.400^∗∗^	


*BBC, Belief in Climate Change; CCRP, Climate Change Risk Perception; KCCA, Knowledge of Climate Change Action; PICC, Perceived Intractability of Climate Change; CCI, Climate Change Inaction. ^∗^*p* < 0.05; ^∗∗^*p* < 0.01.*

**Table 5:** Means (SDs) and independent samples *T-*test for major variables of Study 3.

	Individualist group	Collectivist group	*t*
		
	*M* (S*D*)	*M* (*SD*)	
Individualistic orientation	58.02 (7.68)	49.32 (11.06)	5.460^∗∗^
Collectivistic orientation	49.80 (10.07)	59.94 (8.98)	–6.307^∗∗^
BCC	8.31 (2.16)	8.87 (2.04)	–1.656
CCRP	23.97 (4.45)	24.07 (5.79)	–0.867
KCCA	4.42 (1.96)	5.02 (2.37)	–1.664
PICC	13.69 (4.33)	10.87 (4.64)	3.815^∗∗^
CCI	3.24 (1.64)	2.39 (0.96)	3.682^∗∗^


*BBC, Belief in Climate Change; CCRP, Climate Change Risk Perception; KCCA, Knowledge of Climate Change Action; PICC, Perceived Intractability of Climate Change; CCI, Climate Change Inaction. ^∗^*p* < 0.05; ^∗∗^*p* < 0.01.*

An ANCOVA was conducted with BCC, CCRP, and KCCA as co-variates. The effect of group remained significant for PICC [*F*(1,151) = 10.150, *p* < 0.01, $\eta_{p}^{2}$ = 0.063] and CCI [*F*(1,151) = 12.447, *p* < 0.01, $\eta_{p}^{2}$ = 0.076]. These results show that the differences in PICC and CCI between the two groups were independent of BCC, CCRP, and KCCA. Participants in the individualist group reported higher levels of PICC, *t*(154) = 3.87, *p* < 0.001, *M*_d_ = 2.82, 95% CI = [1.38, 4.26], Cohen’s *d* = 0.64, and higher levels of CCI, *t*(154) = 3.68, *p* < 0.001, *M*_d_ = 0.85, 95% CI = [0.39, 1.31], Cohen’s *d* = 0.61, than did those in the collectivist group.

To further investigate whether PICC has a mediating role between participants’ individualist/collectivist status and CCI, a mediation analysis was performed with CCI as the dependent variable, individualist/collectivist status as the independent variable, and PICC as the mediator. In addition, BCC, CCRP, and KCCA were treated as control variables. For this, M-plus 7.4 was used with 5,000 bootstrap samples (Preacher and Hayes, 2008). Individualist/collectivist status was dummy coded with collectivist = 0 and individualist = 1, and all variables were standardized prior to analysis so that the results would provide standardized coefficients. Individualist/collectivist status had a significant effect on CCI (*b* = 0.204, *p* = 0.006) and PICC (*b* = 0.294, *p* < 0.001). Additionally, PICC positively predicted CCI (*b* = 0.262, *p* = 0.002). As hypothesized, the tests of indirect effects demonstrated that there was a significant indirect effect of individualist/collectivist status on CCI through PICC (99% CI [0.008, 0.174], *p* = 0.018).

### Discussion

The results of Study 3 indicate that individuals with a more individualist orientation rated climate change more intractable and reported a greater incidence of climate change inaction than did individuals with more collectivist orientation. Further data analysis showed that the perceived intractability plays a mediating role in the relationship between individualist/collectivist status and climate change inaction. The findings were consistent with our hypothesis. Therefore, we infer that there are individualist–collectivist differences in climate change inaction, which may contribute to the level of perceived intractability of climate change.

## General Discussion

A central focus of the present article was to examine whether individualists would report a greater incidence of climate change inaction due to higher perceived intractability than collectivists. For this purpose, three studies were conducted. The main findings can be readily summarized. Study 1 showed that participants’ self-reported frequency of climate change action in the preceding 6 months was negatively related to their perceived intractability. In other words, the more intractable participants felt climate change to be, the more demotivated they were to take climate change action. This result was supported in Study 2, which suggested that participants exposed to information concerning the intractability of climate change showed a significantly greater perceived intractability of climate change and lower intentions to assume a low-carbon lifestyle than those presented with neutral information. Based on the first two studies, participants with a collectivist or individualist orientation were recruited from a pool of Chinese undergraduate students in Study 3. We found that participants with more individualist orientations were more subject to perceived intractability and more likely to demonstrate climate change inaction than those with more collectivist orientations. Moreover, the mediating role of perceived intractability in relations between individualist/collectivist status and climate change inaction was also confirmed.

The present findings may contribute to increasing our understanding of climate change inaction. The critical role of psychological barriers in climate change has been underlined by the recent researches (Gifford, 2008). Existing theories and empirical studies have proposed a wide variety of psychological barriers that trigger climate change inaction (Frantz and Mayer, 2009; Aitken et al., 2011; Salomon et al., 2017). However, the literature is still limited on how the latent psychological barriers incur climate change inaction. In this article, we argue that the construct of perceived intractability provides a useful perspective from which to examine this matter. Without doubt, climate change is intractable in itself because greenhouse gas emission cannot be drastically reduced by the efforts of scattered individuals. This perceived intractability of climate change may further induce individuals to remain inactive against climate change. Our work is the first to show that the incidence of climate change inaction is indeed related to perceived intractability even when other related variables were controlled for. This result is in line with findings reported by previous literature. Aitken et al. (2011) found that stronger perceptions of powerlessness were related to lower levels of action to mitigate climate change. Likewise, Salomon et al. (2017) suggested that energy conservation behavior was connected with climate change helplessness-the belief that one’s actions cannot affect climate change. In this sense, perceived intractability offers another avenue of insight to help us to understand why we are so reluctant to take action against climate change.

Clearly, climate change inaction is not a homogeneous phenomenon, and hence investigating individual differences in climate change inaction should contribute to a better understanding of why and how this phenomenon occurs. Individualism–collectivism-based differences in pro-environmental behavior have been reported in earlier studies (Mccarty and Shrum, 2001; Cho et al., 2013), which provided the impetus for this investigation of individualism–collectivism-based variations in climate change inaction. Consistent with existing findings, our results demonstrated that collectivist orientations may be more related to climate-friendly behaviors than are individualist orientations. Not only this, we further showed that these differences could be attribute to the perceived intractability of climate change. Extensive evidence indicates that our responses to climate change could be shaped by cultural orientations (Heyd and Brooks, 2009; Hoffman, 2010) at both the attitudinal and behavioral levels. The significance of our results lies in the fact that they make a unique contribution to the existing knowledge about climate change inaction by showing that individualism–collectivism shapes barriers to perform climate-friendly behaviors.

The present findings may shed some light on nudging public engagement in climate change. Public engagement, as one critical aspect in addressing climate change, has been repeatedly emphasized in public policy agendas worldwide. From an instrumental perspective, climate change is in no small part due to human activity, or more accurately, innumerable individual activities. Thus, any policy or action aimed at climate change mitigation and adaptation will largely depend on public support and participation. However, the resounding calls for public engagement raises one challenge for academics and practitioners, namely, how to promote public engagement in climate change (Whitmarsh et al., 2013; Van et al., 2015). In line with Campbell (1983), climate change is not intractable in itself given the potential efforts of every member of human society. Nevertheless, the results of the present paper suggest that perceived intractability may induce climate change inaction. Thus, it is inadvisable to rush to calls for public engagement in fighting climate change; instead, policymakers should encourage the public to believe that their individual actions are necessary as well as efficacious. More importantly, risk communication in the context of climate change should inform the public that climate change is not just potentially catastrophic, but solvable. This promising tactic is specific to special groups with individualist orientations, as they are more inclined to view climate change as intractable.

The present findings may also be of special significance to climate change mitigation in Chinese cultural context. As we all know, China is the world’s largest carbon dioxide emitter. According to Global Carbon Budget, 2017 published by the Global Carbon project, China covered 28% of global emissions in 2017 (Global Carbon Budget, 2017). This big number may be hard to plummet, due to China’s pursuing economic growth. What is more, conspicuous consumption and materialism are emerging in contemporary China (Podoshen et al., 2011; Sun et al., 2014, 2017), which may in turn hinder individuals to perform low-carbon lifestyles. In this case, policymakers and researchers will be confronted with a tough issue of promoting public engagement in climate change. On the other hand, there is enough evidence to indicate that individualism is increasing and collectivism is decreasing in contemporary China (Yang, 1996; Yi and Takeshi, 2014; Cai et al., 2018). As indicated by our findings, the rise of individualism may further pose a challenge for public engagement in climate change, because individualism is more related with climate change inaction. The above-mentioned fact should not be interpreted with pessimism. Instead, it reminds us that public engagement in climate change is possible only when psychological barriers of climate change inaction are overcome.

Nevertheless, some aspects of the current research require further consideration. First, the measures of belief in climate change used in the present study have face validity, but the internal consistency was lower than optimal. Although belief in climate change was treated as an irrelevant variable, study results may still be swayed by this flaw, suggesting that our findings should be interpreted cautiously. Second, the individualism–collectivism orientation was examined only within Chinese cultural background. To generate more conclusive support for the individualist–collectivist differences in climate change inaction, research conducted in other cultural backgrounds or using a cross-cultural comparative approach is necessary. Third, another limitation of the present studies is that we used self-report measures rather than measures of actual climate change (in)action. For future research, it is worthwhile to examine climate change inaction with objective measurements related to a low-carbon lifestyle. Fourth, given that examining individualist–collectivist differences in climate change inaction was the original goal of the present research, an intervention study was not conducted. Meleady and Crisp (2017) redefined climate change inaction as temporal intergroup bias, and found that temporally adapted interventions for reducing prejudice may help elicit environmental protection. Similarly, future studies are expected to investigate how to encourage climate change action by overcoming the perceived intractability of climate change. Fifth, future studies could explore the boundary conditions of climate change inaction. Although, we distinguished intractability from helplessness, with an emphasis on the view that climate change is inherently intractable whereas helplessness may be a subjective experience concerning climate change, it is possible that perceived intractability make people feel helpless, which in turn results in climate change inaction. Therefore, it will be important to further explore the mediating role of helplessness in the relation between perceived intractability and climate change inaction. Moreover, the effect of perceived intractability on climate change inaction may be moderated by collective efficiency. Collective efficiency, people’s shared belief in their collective power to produce desired results (Chen, 2015), may act as a buffer against the perceived intractability of climate change and further promote climate-friendly behavior.

## Ethics Statement

This research is approved by the Institutional Review Board of Nanjing University. All subjects gave written informed consent in accordance with the Declaration of Helsinki. The protocol was approved by the Institutional Review Board of Nanjing University.

## Author Contributions

PX designed the study and drafted the manuscript. PX and HZ did the majority of the work on data collection and data analysis. LG contributed to the conception and design of the work. LG, KZ, and YW polished the manuscript.

## Conflict of Interest Statement

The authors declare that the research was conducted in the absence of any commercial or financial relationships that could be construed as a potential conflict of interest.

## Footnotes

1 Similiar grouping procedures are common in research related to individualism/collectivism, especially those conduced within a single cultural background. In these studies, participants were artificially categorized as individualists or collectivists based on their scores on an instrument (e.g., Liddell et al., 2015) or through individualism/collectivism priming (e.g., Goncalo and Staw, 2006). Despite latent limitations, the individualism/collectivism dichotomy could methodologically avoid a situation in which individuals simultaneously exhibit individualist and collectivist orientations.

## Appendix 1: Passage Used for Study 2.

**Experimental Group** Considering excessive carbon emission Is one of the larger contributors to climate change, it Is possible to lower carbon emission via reducing energy consumption in order to tackle climate change. According to a report published by the Global Carbon Project, global carbon emissions in 2013 reach a record high of 36 billion tons, and this number Is still growing. However, the amounts of carbon emissions reduced by a single individual seem to Be rather limited. for example, it Is estimated that if we Can decrease the time for having the television on by 1 h each day, the amount of carbon emission May Be reduced by 4.71 kg each month. **Control Group** Global warming Has Been identified as one of the most pressing issues human Is facing. Global warming occurs because of greenhouse gases effect, that is, atmospheric concentration of greenhouse gases (i.e., carbon dioxide) increases sharply due to human activities, and further absorb large amounts of the infrared radiation From the Earth, hence resulting in the rise of average global temperature. In order to obtain the energy, human burn fossil fuel such as oil, coal, and natural gas, which in turn emit an overwhelming quantity of greenhouse gases Into the atmosphere. Furthermore, forests’ role as absorbers of carbon dioxide Has Been impaired by large-scale deforestation.
